# Mechanical Loading Attenuates Radiation-Induced Bone Loss in Bone Marrow Transplanted Mice

**DOI:** 10.1371/journal.pone.0167673

**Published:** 2016-12-09

**Authors:** Peter M. Govey, Yue Zhang, Henry J. Donahue

**Affiliations:** 1 Division of Musculoskeletal Sciences, Department of Orthopaedics and Rehabilitation, Penn State College of Medicine, Hershey, PA, United States of America; 2 Department of Biomedical Engineering, Penn State College of Engineering, University Park, PA, United States of America; 3 Department of Cellular and Molecular Physiology, Penn State College of Medicine, Hershey, PA, United States of America; 4 Department of Biomedical Engineering, Virginia Commonwealth College of Engineering, Richmond, VA, United States of America; University of Sheffield, UNITED KINGDOM

## Abstract

Exposure of bone to ionizing radiation, as occurs during radiotherapy for some localized malignancies and blood or bone marrow cancers, as well as during space travel, incites dose-dependent bone morbidity and increased fracture risk. Rapid trabecular and endosteal bone loss reflects acutely increased osteoclastic resorption as well as decreased bone formation due to depletion of osteoprogenitors. Because of this dysregulation of bone turnover, bone’s capacity to respond to a mechanical loading stimulus in the aftermath of irradiation is unknown. We employed a mouse model of total body irradiation and bone marrow transplantation simulating treatment of hematologic cancers, hypothesizing that compression loading would attenuate bone loss. Furthermore, we hypothesized that loading would upregulate donor cell presence in loaded tibias due to increased engraftment and proliferation. We lethally irradiated 16 female C57Bl/6J mice at age 16 wks with 10.75 Gy, then IV-injected 20 million GFP(+) total bone marrow cells. That same day, we initiated 3 wks compression loading (1200 cycles 5x/wk, 10 N) in the right tibia of 10 of these mice while 6 mice were irradiated, non-mechanically-loaded controls. As anticipated, before-and-after microCT scans demonstrated loss of trabecular bone (-48.2% Tb.BV/TV) and cortical thickness (-8.3%) at 3 wks following irradiation. However, loaded bones lost 31% less Tb.BV/TV and 8% less cortical thickness (both p<0.001). Loaded bones also had significant increases in trabecular thickness and tissue mineral densities from baseline. Mechanical loading did not affect donor cell engraftment. Importantly, these results demonstrate that both cortical and trabecular bone exposed to high-dose therapeutic radiation remain capable of an anabolic response to mechanical loading. These findings inform our management of bone health in cases of radiation exposure.

## Introduction

Exposure to significant levels of radiation increases one’s risk of bone fragility fractures. Space travel and accidental exposure may both result in doses detrimental to bone health, but the most common source is radiation therapy. Purposeful exposure to radiation is an enduring feature of modern medicine despite the ensuing damage to bone tissue. Patients receive localized or total body radiation therapy for a variety of malignancies, blood and marrow-borne cancers and hematological disorders. Unfortunately, this can lead to osteoporosis, osteonecrosis, and elevated fracture risk attributable to depletion of bone marrow cells, reduced bone formation, increased resorption, fatty accumulation, and bone matrix damage [1,2]. Total body irradiation (TBI) is used as part of preparative regimens prior to hematopoietic stem cell (HSC) transplantation in select populations. The extent of long-term bone loss among irradiated HSC (i.e., bone marrow) transplantation recipients is well-characterized in this growing population of long-term survivors [3,4].

We utilized an established mouse model of radiation-induced bone loss from the field of immunology: total body irradiation followed by bone marrow transplantation [5]. Mice were subjected to TBI sufficient to deplete bone marrow cells, including immune cells and hematopoietic stem cells, as was well as osteoblasts and mesenchymal stem cells. In this model trabecular bone volume decreases within days in a radiation dose-dependent fashion. Cortical bone alterations, mainly endocortical resorption, are more subtle. One approach for preventing radiation-induced bone loss is bisphosphonate therapy [6–8]. Denosumab treatment in an adolescent [9] and hyperbaric oxygen therapy in mice [10] have also helped minimize loss of bone viability. Intermittent PTH alleviates localized radiation-induced trabecular bone loss in rodents via a prosurvival effect on osteoblasts and osteocytes [11–13].

Using this TBI/bone marrow transplantation model, we examined the effects of a non-pharmacological intervention: dynamic mechanical loading. In vivo compression loading of mouse tibias increases both trabecular and cortical bone mass [14–16]. Similarly, mouse tibias compression loaded 5 months after a localized, space travel-pertinent dose of 2 Gy heavy ion irradiation also demonstrated increased periosteal bone formation rates [17]. However, this irradiation had no observed effect on bone microstructure and transient changes in bone cell populations were far removed. No known studies have assessed mechanical loading in the immediate aftermath of therapeutic radiation doses. We hypothesized that in vivo compression loading attenuates bone loss in irradiated, bone marrow transplanted mice, as evident by changes in trabecular and cortical microstructure assessed by microCT.

Incorporation of systemically-injected cells in both damaged and healthy bone is of wide interest. It is apparent that some pathology or conditioning must be present for labelled cells to engraft within bone. Mouse models of osteogenesis imperfecta form bone from cells systemically injected in neonates [18] and locally injected in adults [19] after priming marrow with sub-lethal irradiation. Fractures [20–22] and calvarial defects [23] also recruit IV-injected cells. However, compression loading in otherwise healthy mice did not yield engraftment of IV-injected MSCs at sites of loading-induced bone formation [24]. Total body irradiation is an impetus for host engraftment of hematopoietic stem/progenitor donor cells to bone marrow, and efficient hematopoietic recovery dictates survival. HSCs and mesenchymal osteolineage cells share a mutually-regulated endosteal, perivascular niche [25–28]. Molecules up-regulated in bone by mechanical loading, including CXCL12/SDF-1 [29] and PGE_2_ [30], increase marrow engraftment and recovery of donor hematopoietic cells [31–34], yet the influence of load-induced signaling is unknown. We further hypothesized that mechanical loading of long bones in an irradiation-primed context up-regulates engraftment and proliferation of donor-derived cells.

We report loss of trabecular bone and endosteal cortical bone following irradiation and bone marrow transplantation. Analysis of donor cell presence in loaded vs. non-loaded tibias revealed no significant increase in total marrow cell proportion or donor DNA in cortical bone, though there was an apparent trend of increased donor DNA in loaded bones. However, we did find that mechanical loading attenuated radiation-induced bone loss.

## Materials and Methods

### Irradiation and bone marrow transplantation

Young adult C57Bl/6J mice (16 females, age 16 wks, purchased from the Jackson Laboratory, Bar Harbor, ME) were treated with 600+475 cGy (67.5 cGy/min) 3-hour-split doses of whole body irradiation in an X-RAD 320 biological irradiator (Precision X-Ray, North Branford, CT) equipped with an F2 beam conditioning filter. This myeloablative dose was lethal without donor bone marrow inoculation. Syngeneic donor bone marrow was flushed, filtered by 70 μm nylon mesh, and pooled from femurs and tibias of at least 2 male 10 wk old C57Bl/6-Tg(CAG-EGFP)131Osb/LeySopJ mice [35] (Jackson Laboratory) using phosphate buffered saline (PBS) + 5% embryonic stem cell-grade fetal bovine serum (ES-FBS) [Gibco]. Virtually all tissues except hair and red blood cells (RBCs) express enhanced GFP in these donor mice [35]. Within 24 hours of irradiation, a 150 μL suspension of PBS+5% ES-FBS with 20 million whole bone marrow cells was intravenously injected [36] at the retro-orbital sinus [37] of recipient mice under isoflurane anesthesia after pre-treating mice with 0.5% proparacaine hydrochloride ophthalmic analgesic solution (Alcon Laboratories).

Immunocompromised mice were maintained in sterile housing with acidified water, wetted food *ad libitum*, and 1 post-irradiation week with DietGel Recovery nutritional supplement (ClearH_2_0, Portland, ME) and antibiotic-supplemented water (Sulfamethoxazole and Trimethoprim oral suspension, 200 mg/40 mg per 5 mL). Cages were opened within BSL-2 safety cabinets. During open-air compression loading, clean personal protective clothing was worn, including sterile gloves and face mask. Surfaces of loading and anesthesia apparatus were sterilized with MB-10 disinfectant solution (Quip Laboratories). Animal protocols were approved by Penn State College of Medicine IACUC #42521 with appropriate supportive care as recommended by Duran-Struuck and Dysko [5].

### *In vivo* microcomputed tomography

Within 12 hours of irradiation, tibias of live animals were scanned for baseline microarchitecture in randomized sequence. Scans were not possible prior to irradiation because of sterile facility restrictions. Tibias were evaluated because of the capacity to externally load these bones. Right and left limbs, consecutively, were positioned in a Scanco rodent hindlimb immobilization device for scanning using a Scanco vivaCT 40 (Scanco Medical AG, Brüttisellen, Switzerland) while under 2% isoflurane anesthesia at 1 L/min. MicroCT scans were repeated in vivo at the completion of 3 weeks. We quantified trabecular parameters from a 74-slice region of the proximal tibia immediately distal to the epiphyseal plate, and cortical parameters from a 24-slice region immediately proximal from the mid-shaft and roughly 2 mm proximal from the tibio-fibular junction. Each region was composed of 10.5 μm isotropic voxels with instrument settings of 55 kVp, 145 μA, and 200 ms integration time. Images were Gaussian filtered (sigma = 1.5, support = 2) and a threshold (27.5% of full scale) was applied to remove the surrounding soft tissue.

Trabecular bone was manually segmented with the aid of the Scanco morphing algorithm. As per published guidelines [38], trabecular parameters included bone volume fraction (BV/TV), number (Tb.N), thickness (Tb.Th), separation (Tb.Sp), structure model index (SMI), connectivity density (Conn.D), tissue mineral density (Tb.TMD), and degree of anisotropy (DA).

Cortical cross-sectional measurements are sensitive to misalignment of the tibia and the z-axis of the scanner. Compression loading may reduce extension of the knee joint for scan positioning, thereby biasing cortical measurements. Still, *in vivo* post-scans were necessary since subsequent bone marrow analysis precluded *ex vivo* scans. Therefore, we applied Scanco’s *align in z and reimport* command with the cubic interpolation option to align all pre and post cortical scan slices orthogonal to the central intramedullary axis of the cortical volume. Periosteal and endosteal boundaries of the cortical bone were segmented using the Scanco semi-automated edge detection algorithm. Cortical parameters included total area (Tt.Ar) enclosed by the periosteum, cortical area (Ct.Ar) excluding marrow cavity, area fraction (Ct.Ar/Tt.Ar), marrow area (Ma.Ar), cortical thickness (Ct.Th), porosity (Ct.Po), and cortical bone mineral density (Ct.BMD).

For structural indices, we calculated each bone’s percent change from baseline. S1 Fig and S2 Fig also present raw trabecular and cortical data as scattered dot plots. Images of 3D trabecular and cortical morphology were collected from representative animals demonstrating median changes in trabecular bone volume fraction or cortical area.

### Mechanical Loading

We initiated a non-invasive axial loading regimen of the right tibia immediately prior to inoculation and continuing 5 days/wk for 3 wks. A typical axial loading protocol for mice is detailed by Melville and colleagues [16]. The right leg of anesthetized, supine mice was positioned horizontally in line with an aluminum cup against the flexed knee and a rigid plastic fixture holding the flat foot at 30 degrees dorsiflexion similar to Fritton, et al [14]. Tibias were loaded for 1,200 cycles/day with 10 N compression in a sawtooth waveform at 4 Hz, including 0.1 s dwell at 2.5 N between cycles. Previous strain gauge analysis under identical dynamic loading in female 16-week-old C57Bl/6J mice demonstrated a linear load-microstrain (μϵ) relationship between a series of load magnitudes applied at the medial mid-shaft (3.8, 6.3, 8.6, and 11.3 N) [39]. Based upon a load of 11.3 N corresponding with tibial deformations of about 1850 μϵ at our site of mid-shaft scanning [39], we interpolate our applied 10 N imparted approximately 1640 μϵ upon initiation of loading. A control group of 6 mice was concurrently anesthetized but not loaded. Both groups received IP injections of 0.075 mg/kg buprenorphine during treatments to minimize any discomfort and encourage normal ambulation.

### Bone marrow analysis

After day 21 microCT post-scans, mice were subjected to a loading bout and then sacrificed by CO_2_ asphyxiation and cervical dislocation. Tibias were immediately dissected from both hind limbs and placed in PBS solution on ice. In a cell culture hood, the proximal and distal epiphyses were cut with a scalpel, followed by a cut at approximately 1/3 of the length from the proximal end. This cut was consistently made at the apex of the anterior ridge of the tibia. Each piece was returned to separate dishes containing PBS with 5% ES-FBS and marrow was flushed and strained with a 25g needle. Cells were filtered by nylon mesh and counted using a TC10 automated cell counter (Bio-Rad) followed by live-cell flow cytometry analysis using a FACSCanto II (BD Biosciences). Populations of single cells were gated using side scatter vs. forward scatter dot plots, and GFP expression was gated based upon FITC fluorescence detection using 488 nm excitation and emission at 530 nm. Prior to flow cytometry, separate cell aliquots were incubated with 1x RBC lysis buffer for 10 minutes at room temperature, mixing well. 10x RBC lysis buffer consisted of 1 L dH_2_0 + 80.2 g NH_4_Cl + 8.4 g NaHCO_3_ + 3.7 g EDTA disodium. Cells were centrifuged for 10 minutes at 1200 RPM, aspirated, resuspended in PBS+ES-FBS, and filtered for flow cytometry analysis.

### Relative DNA quantification

We analyzed relative DNA of donor cell-specific genes from proximal and distal tibia bone segments. Immediately after marrow was flushed from bone segments, each segment, epiphyses removed, was snap-frozen in liquid nitrogen, placed in unique labeled tubes, and stored at -80°C for later processing. Some bones were fragmented during bone marrow removal, and these fragments were included with the frozen sample. Using a DNeasy blood and tissue kit (Qiagen, Valencia, CA), bone segments were thawed in lysis buffer and mineral was homogenized with a rotor-stator homogenizer and incubated in a shaker bath at 56°C overnight. Samples were vortexed, then centrifuged to separate powdered mineral, and aspirate was processed in spin columns to isolate DNA according to Qiagen’s instructions. Quantitative PCR for both the GFP transgene and male Y chromosome-specific *Zfy1* [40] was then carried out in triplicate with the QuantiTect SYBR-Green PCR kit (Qiagen, Valencia, CA) and normalized to *ApoB*. Murine-specific primers are specified in Table 1. Specificity of PCR products was confirmed by gel electrophoresis. Bones from wild-type female host mice without donor cell injection did not demonstrate amplification of either gene. Relative expression levels for each bone segment were calculated as 2^(Ct[*ApoB*]-Ct[*GFP*,*Zfy1*]) [41].

**Table 1:** Murine-specific primers used for real-time RT-PCR for relative quantification of donor cell DNA.

Gene	Primer Description	Sequence (5’ to 3’)
**GFP transgene** (Jackson Labs)	Forward (primer 10201)	AGTGCTTCAGCCGCTACC
	Reverse (primer 10202)	GAAGATGGTGCGCTCCTG
***Zfy1*** [40]	Forward	TGGAGAGCCACAAGCTAACCA
	Reverse	CCCAGCATGAGAAAGATTCTTC
***ApoB*** (Jackson Labs)	Forward (primer oIMR1544)	CACGTGGGCTCCAGCATT
	Reverse (primer oIMR3580)	TCACCAGTCATTTCTGCCTTTG

### Statistical analysis

We conducted statistical analyses using GraphPad Prism 6 (La Jolla, CA). Data are presented as mean ± 95% confidence interval (CI). Differences between two groups were compared using a Student’s *t*-test pairing loaded and contralateral tibias, and more than two groups were compared using repeated measures one-way ANOVA with Tukey’s multiple comparisons test. Where applicable, we used repeated measures two-way ANOVA for group comparisons (control/loaded and proximal/distal). For all comparisons, a *p* value <0.05 was considered significant.

## Results

### Irradiation tolerance

**Fig 1:**
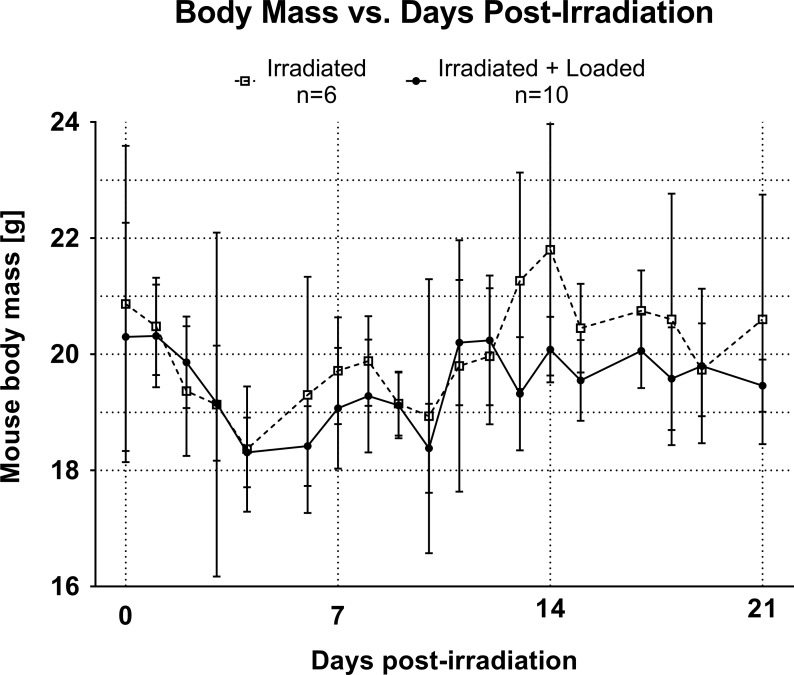
Mouse body mass as a function of time, from irradiation treatment on day 0 to sacrifice on day 21. Solid line, filled circles represent ‘Irradiated + Loaded’ animals and dashed line, open squares represent ‘Irradiated’ animals. Mean ± 95% CI, n = 3–10 for each data point due to intermittent weighing.

All mice survived to the 3-week endpoint, though initial sickness evident by body mass loss and recovery (Fig 1) was noted, as anticipated following irradiation. Maximal weight loss from baseline, occurring at 4 days post-irradiation, was -9.8% for loaded mice and -12.0% for non-loaded controls. At 21 days, final average weight loss from baseline was -4.1% for loaded mice and -1.3% for non-loaded controls. Differences between groups were not significant.

### Trabecular bone changes

**Fig 2:**
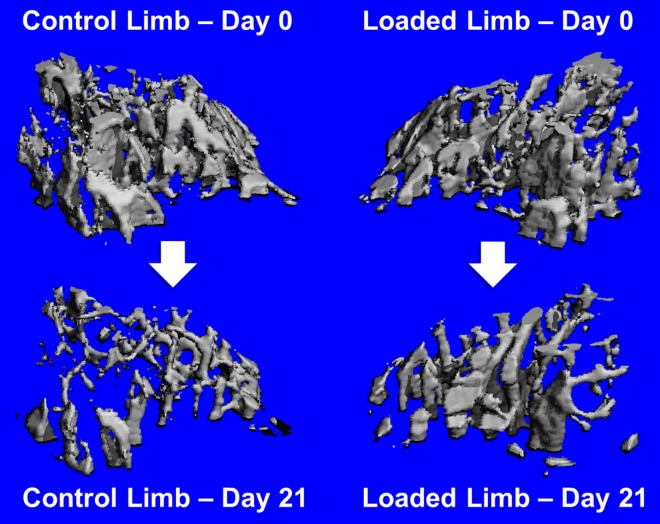
Representative trabecular bone changes from one mouse having median changes in bone volume fraction.

**Fig 3:**
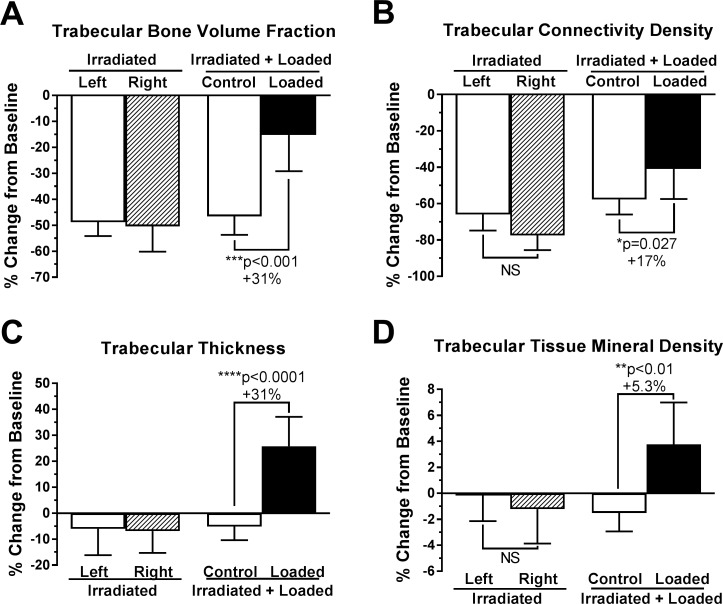
Trabecular microstructure parameters significantly regulated by loading, as indicated by microCT. Mean ± 95% CI, n = 10 for ‘Irradiated+Loaded’ mice and n = 6 for ‘Irradiated’ mice. p<0.05 considered significant by paired two-tailed t-test. Percent changes indicate effect of loading relative to contralateral controls.

Irradiation treatment resulted in significant deterioration of trabecular bone quality as evident visually (Fig 2) and by various structural parameters (Fig 3). Additional raw data is available as scattered dot-plots in S1 Fig. Relative to baseline, among all non-loaded tibias irradiation decreased trabecular bone volume fraction by -48.2±3.8%, nearly half of initial trabecular bone. Connectivity density decreased by -65.3±5.6%, trabecular thickness by -5.9±3.5%, tissue mineral density by -1.1±0.9%, trabecular number by -23.4±2.6%, and degree of anisotropy by -8.4±3.3%. Irradiation increased trabecular separation by +32.1±4.5% and the structure modeling index, an indicator of deterioration from “plate-like” to “rod-like” architecture [42], increased +31.2±4.9% to become more rod-like. All changes were similar among left and right limbs of non-loaded mice and internal control limbs of loaded mice. Trabeculae are fewer, thinner, farther apart, and less dense.

**Fig 4:**
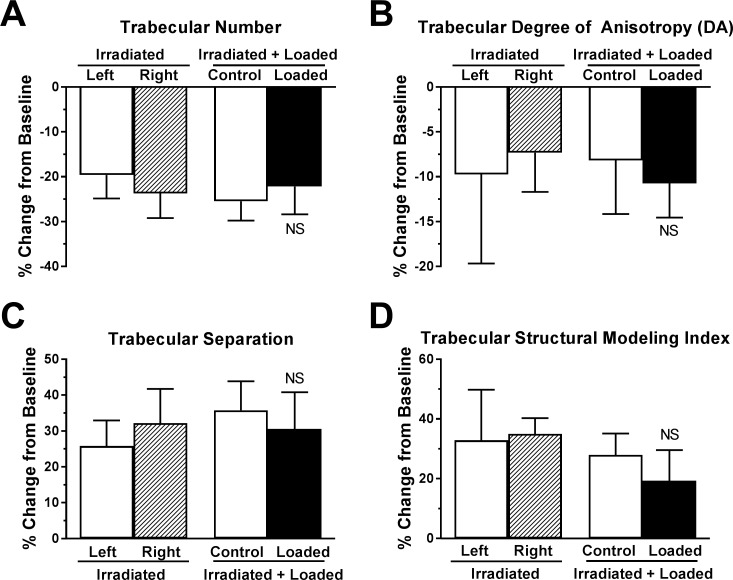
Additional trabecular microstructure parameters not significantly regulated by loading, as indicated by microCT. Mean ± 95% CI, n = 10 for ‘Irradiated+Loaded’ mice and n = 6 for ‘Irradiated’ mice. p<0.05 considered significant by paired two-tailed t-test. NS indicates no significant difference between contralateral controls.

However, loading significantly attenuated trabecular bone loss. Relative to baseline, loaded bones lost only -15.2±13.9% of bone volume fraction and -41±16.5% of connectivity density (Fig 3). Trabecular thickness *increased* by +25.8±11.3% from baseline, and tissue mineral density also increased +3.8±3.2%. Thickening of persistent individual trabeculae was observed in microCT reconstructions, of which Fig 2 is typical. Remaining trabecular parameters were not significantly regulated by loading (Fig 4).

### Cortical bone changes

**Fig 5:**
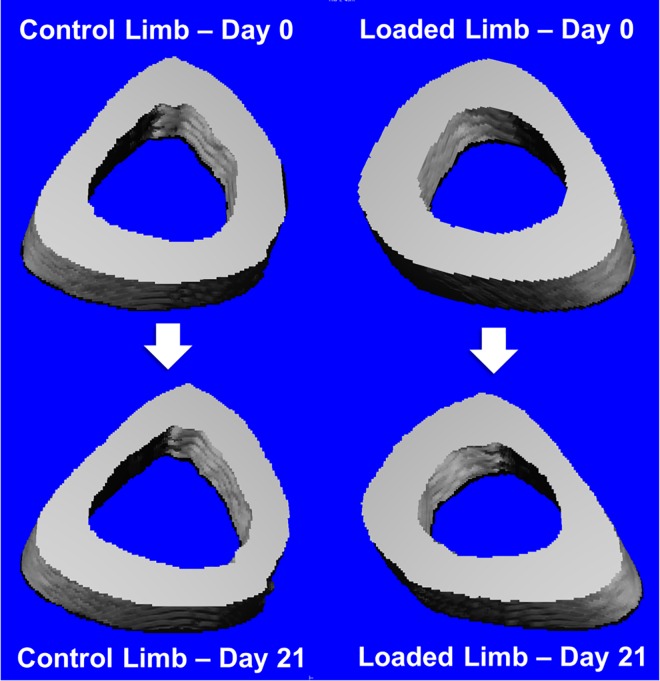
Representative cortical bone changes from one mouse having median changes in cortical area.

**Fig 6:**
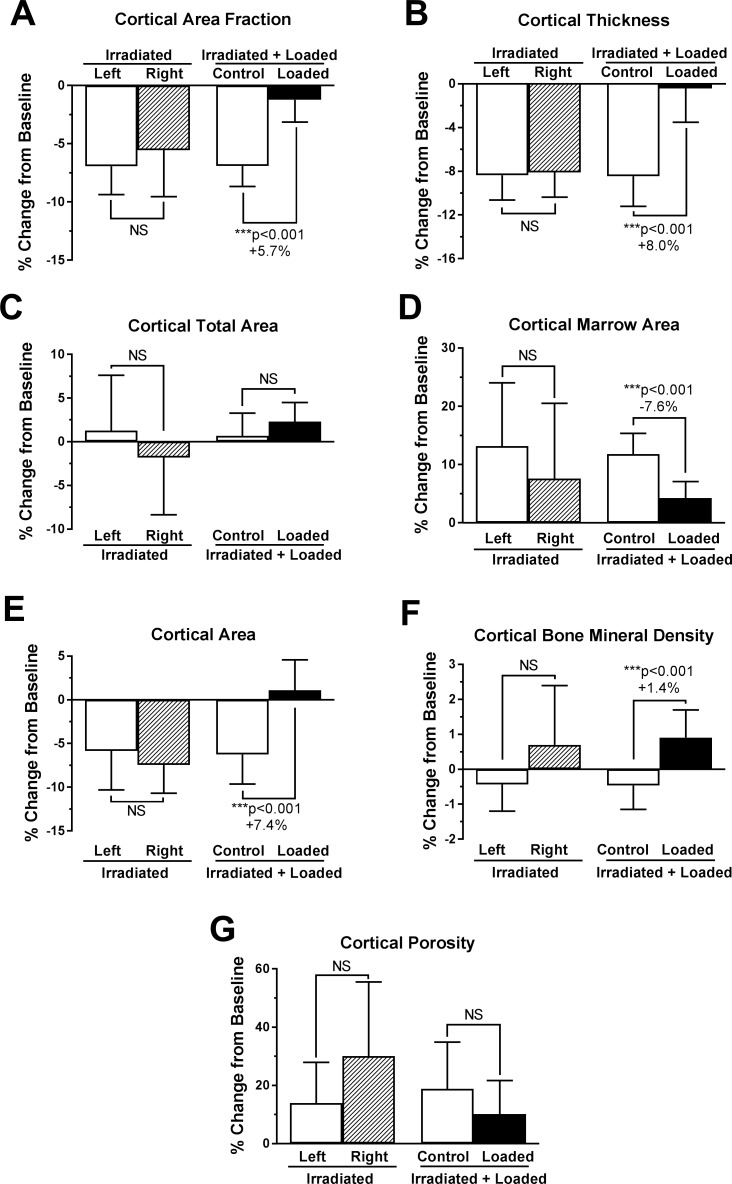
Cortical microstructure parameters significantly regulated by loading, as indicated by microCT. Mean ± 95% CI, n = 10 for ‘Irradiated+Loaded’ mice and n = 6 for ‘Irradiated’ mice. p<0.05 considered significant by paired two-tailed t-test. Percent changes indicate effect of loading relative to contralateral controls.

Though cortical bone changes are visually subtle (Fig 5), cortical parameters here at the midshaft degraded following irradiation, as well (Fig 6, S2 Fig). Among all non-loaded tibias, the cortical area and cortical area fraction were decreased by -6.5±1.8% and -6.5±1.2%, respectively, relative to baseline. This is attributable to reduced average cortical thickness (-8.3±1.3%) and increased marrow area (+11.0±3.9%) with no change in total area (0.2±2.2%). Essentially, irradiated bones lost mostly endosteal bone.

Loaded bones, however, lost less endosteal bone and maintained cortical thickness (Fig 6). Relative to baseline, marrow area increased only 4.2±2.9% and the result is attenuated loss of cortical area fraction from baseline (-1.2±1.9%). Additionally, average cortical thickness held steady (-0.4±3.1%) as did cortical area (+1.1±3.5%). The effect of loading relative to non-loaded contralateral limbs was +5.7% greater cortical area fraction, +8.0% greater cortical thickness, -7.6% less expansion of marrow area, +7.4% greater cortical area, and +1.4% greater cortical bone mineral density.

### Donor cell engraftment and bone marrow analysis

**Fig 7:**
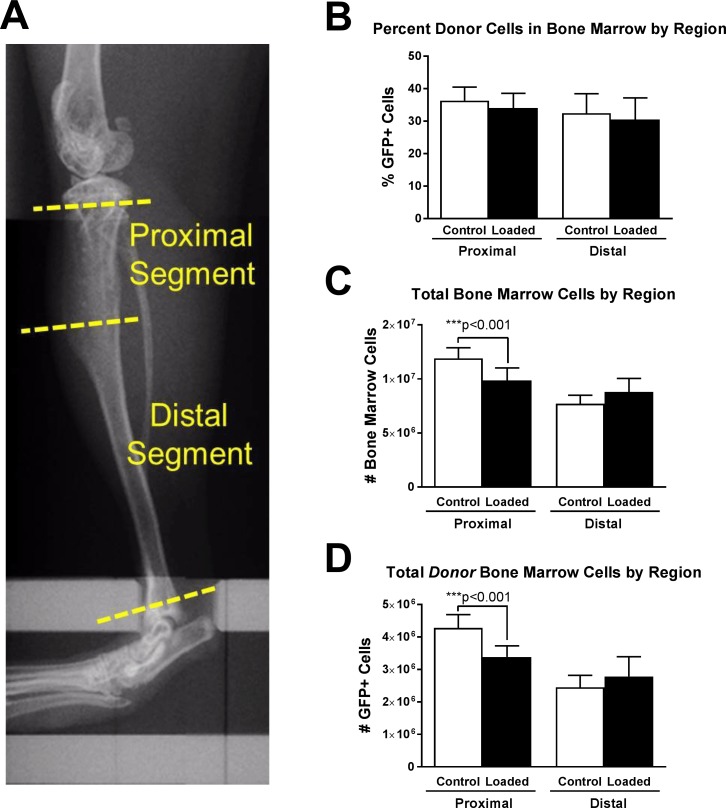
Bone marrow donor/host analysis by tibia region. (A) In vivo microCT scout image indicating locations of cuts made during dissection to isolate proximal trabecular compartments and distal cortical compartments. (B) Percent of freshly isolated total bone marrow cells analyzed as GFP(+) by flow cytometry analysis. Mean ± 95% CI, n = 9–10 mice, all with loaded and contralateral control limbs. (C) Cell count of freshly isolated total bone marrow cells. Mean ± 95% CI, n = 10. (D) Estimated total donor bone marrow cells calculated as each sample’s total bone marrow cell count multiplied by that sample’s percent GFP+ marrow (B x C = D). Mean ± 95% CI, n = 9–10. Significant differences indicated by paired two-tailed t-test.

We used live cell fluorescence-activated flow cytometry to analyze the proportion of GFP-expressing donor bone marrow cells in proximal 1/3 and distal 2/3 of each tibia. These divisions, shown in Fig 7A, approximate respective trabecular and cortical bone compartments. Marrow from all transplanted mice demonstrated engraftment of GFP(+) cells while GFP was undetectable in marrow from non-transplanted mice (data not shown). Fig 7B shows the fraction of all bone marrow cells fluorescently identified as donor origin GFP(+). Approximately 35% of all bone marrow cells were consistently of donor origin, and there were no significant differences between distal and proximal regions or between loaded and contralateral control limbs. Lysis of red blood cells in aliquoted samples increased this average GFP(+) proportion to approximately 60%, and these samples also demonstrated no inter-group differences (data not shown). Fig 7C shows cell counts of total bone marrow cells (absent of RBC lysis), revealing that significantly fewer cells were flushed from proximal tibias of loaded bones relative to contralateral bones. Loaded proximal marrow spaces were also occupied by greater trabecular BV/TV. Data in Fig 7D, counts of donor origin cells, was inferred by multiplying results in panels B and C.

### Relative donor cell DNA in bone

**Fig 8:**
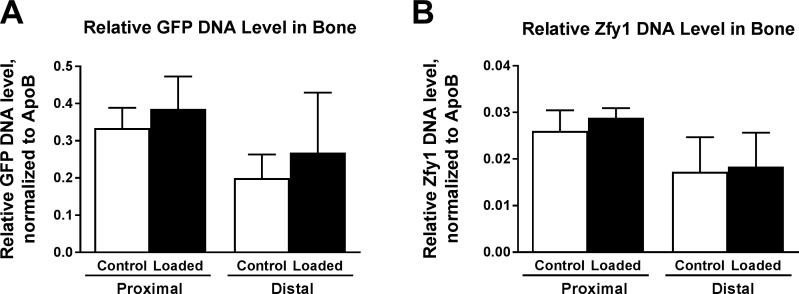
Relative donor cell DNA levels in host bone. Loaded and contralateral control bones each divided by proximal and distal segments. Donor-specific DNA measured by (A) GFP transgene (p = 0.098 for effect of loading and **p = 0.009 for effect of bone region by two-way ANOVA) and (B) male *Zfy1* DNA (p = 0.256 for effect of loading and **p = 0.002 for effect of bone region), both normalized to *ApoB*. Mean ± 95% CI with n = 7 for proximal and n = 5 for distal segments.

After marrow was flushed from proximal and distal tibia segments, we examined relative donor cell DNA levels in bone itself. We analyzed the GFP transgene and Y chromosome-associated *Zfy1*, both of which were specific to donor cells. Normalized relative DNA levels are presented in Fig 8. In both bone regions, we saw a trend of greater donor cell presence in loaded bones, though the effect of loading was not significant by two-way ANOVA (p = 0.098 for GFP and p = 0.256 for *Zfy1*). However, there was a significant effect of bone region for both genes (p = 0.009 for GFP and p = 0.002 for *Zfy1*). Greater donor cell DNA in proximal bone may be due to more residual marrow cell presence following the flushing of marrow. Future histological analysis will be useful for looking at incorporation of donor cells in bone.

## Discussion

The purpose of this study was to examine how mechanical loading influences bone structure and adoption of donor-derived cells following irradiation and bone marrow transplantation. Independent of loading, bone loss experienced by irradiated mice exceeded the loss expected solely with increasing age. Microstructure changes have been well characterized by microCT at the distal femur and corresponding proximal tibia in female C57Bl/6J mice between 16 and 24 weeks of age [43]. Over these two months, bone volume fraction (BV/TV) decreased approximately 40%. Assuming a linear decline, for our 3 week study we would expect about 15% loss of BV/TV due to aging, but we observed nearly 50% BV/TV loss in irradiated limbs. Loss of BV/TV in irradiated + loaded tibias (-15.2%) was in line with that expected due to aging alone. To provide context for this bone loss, male C57Bl/6J, which lose BV/TV more gradually than females between 4–6 months [43], lost about 30–40% BV/TV over 3 weeks due to muscle paralysis unloading [44], and slightly older mice (6 months) lost over 65% BV/TV during 3 weeks of hind-limb suspension unloading [45]. Male 6 wk old C57Bl/6 subjected to irradiation and bone marrow transplantation lost 56% BV/TV relative to age-matched controls in one month, and this deficit persisted through 6 months post-transplantation [46].

As another indicator of irradiation’s effect on bone, trabecular thickness (Tb.Th) decreased about 5% with irradiation despite characteristic increases in 16–24 wk old female mice by 1–2% over 3 weeks [43]. Significant deficits in Tb.Th were not manifested until 6 months post-transplant in younger (6 week) irradiated mice [46]. In cortical bone, we observed average thickness to decrease almost 8% though it characteristically holds steady or slightly increases with age [43].

These cortical and trabecular deficits resulted from a total body radiation dose roughly 15-fold greater than the localized radiation imparted by an in vivo microCT scan. Localized radiation delivered by microCT at each proximal and midshaft tibia scan region was approximately 720 mGy according to Scanco documentation and similar published measurements [47]. MicroCT scans of this dosage every 5–7 days over a period of weeks have led to a small reduction of trabecular parameters in young mice, including a loss of roughly 15% BV/TV and 10% trabecular number, and a trabecular separation increase of 11% [47,48]. The effect of our study’s single baseline microCT scan on bone microstructure should be slight relative to the effect of total body irradiation, but nevertheless additive. Altogether, the total absorbed dose specifically at each region of interest was about 11.5 Gy within the initial day.

In loaded limbs, a variety of trabecular and cortical structural indices supported our hypothesis that compression loading attenuates irradiation-induced bone loss following bone marrow transplantation. No systemic effects of loading [49,50] were observed between non-loaded limbs of loaded and control animals. Intriguingly, Willie, et al. [48] found that mice subjected to 4 microCT scans over the course of compression loading gained even greater Tb.BV/TV than mice scanned once. This result and long-term results from Shirazi-Fard, et al. [17] agree with our finding that irradiated bone maintains the capacity to respond to mechanical loads.

One limitation of this study is that we did not experimentally compare the loading response of irradiated mice relative to non-irradiated mice. Direct comparison may be confounded by changes in bone volume and quality following irradiation [51,52]. Relative to healthy bones, irradiated bones would experience different load-induced strain magnitude and distribution, making it difficult to control for the loading stimulus. However, as a benchmark, healthy mice of corresponding age, strain, and gender were similarly loaded with 8.8 N for 2 weeks, generating loaded tibias with +21% BV/TV, +31% Tb.Th, +3% Tb.TMD, and +17% Ct.Ar relative to non-loaded tibias [53]. These responses are most comparable to our loading’s effect on percent change from baseline: +31% BV/TV, +31% Tb.Th, +5.3% Tb.TMD, and +7.4% Ct.Ar. Another limitation is that correlating bone volume with bone strength is more difficult in irradiated bone than healthy bone [51] due to long-term embrittlement from microdamage accumulation and matrix damage [52,54]. We do not know if loading improves irradiated bone quality in parallel with quantity. Finally, while we studied initially healthy mice, bone metabolism may be further dysregulated by the disease state for which radiation therapy is prescribed [55,56] or by accompanying hormonal or chemo-therapies.

Increases in thickness of trabeculae (26%) and tissue mineral density (3.8%) suggest adaptive bone formation along remaining trabeculae. Thus, while down-regulation of osteoclast resorption might be occurring, new bone formation indicates load-induced osteoblast activation. This is a divergence from the characteristic collapse of osteoblast populations following irradiation [1,2]. Future examination of loading’s impact on osteoblast and osteoclast populations is warranted.

One explanation for irradiated bone’s intact adaptive response is that loading activates or protects endogenous osteoblasts, osteocytes, or osteoprogenitors. Mesenchymal stem cells withstand a higher threshold of irradiation compared with other bone marrow cells [57]. The C57Bl/6 strain is particularly tolerant of whole body irradiation, and also demonstrates robust load-induced bone formation [58]. Perhaps the osteogenic capacity of C57Bl/6, both with and without irradiation, is linked to resilience of their osteoprogenitors. Resilient osteocytes also maintain bone health [59]. Mechanical stimulation activates Wnt/β-catenin signaling [60] in osteocytes, protecting them from glucocorticoid-induced apoptosis [61,62] and associated reactive oxygen species (ROS) [63]. Ionizing radiation provokes apoptotic ROS [64]. PTH similarly protects osteocytes and osteoblasts, and thereby bone, from irradiation [12] through Wnt/β-catenin activation [13]. These are potential therapeutic pathways for mimicking loading’s protective effect on irradiated bone.

An alternative explanation for this intact anabolic response is that intravenously injected donor cells contributed to bone formation. Donor cells reconstituted the bulk population of nucleated bone marrow cells at 3 wks. Our hypothesis was not upheld as there was no difference in donor cell presence between loaded and contralateral bones and marrow compartments, though there was a non-significant trend of increased donor cell DNA in loaded bones. Lineage analysis—both mesenchymal and hematopoietic—and histology may be useful for identifying potential differences in cellular composition and activity. Importantly, direct bone formation by donor cells would demonstrate osteogenic capacity of cells injected in peripheral circulation. In any case, donor cells play an essential supportive role by preventing collapse of hematologic functions, and perhaps specific paracrine and systemic signaling [65,66]. Though sub-lethal or focally-irradiated models [67] do not require cell transplantation, this chimeric model advances our ability to investigate mechanical regulation of exogenous cells in the bone marrow microenvironment.

Clinical implications of our results may be considered in light of general recommendations that cancer patients “avoid inactivity” during and after cancer treatment for improved quality of life and physical function [68]. Allogeneic stem cell transplant recipients share these benefits from exercise [69,70], as well as potentially reduced graft-versus-host disease (GVHD) [71] and greater survival [72,73]. Survival was increased in recipient mice subject to an exercise regimen prior to transplantation, though donor cell engraftment was not affected [74]. Despite the acceptance of physical activity as adjuvant therapy following stem cell transplantation, studies have either not examined bone, or were inconclusive due to poor long-term adherence [75]. Future studies of physical activity interventions should include longitudinal bone health as an outcome for surveillance.

We conclude that bone severely compromised by irradiation is not inexorably in decline, but in fact capable of responding to an anabolic stimulus with appropriate systemic cellular support. In the immediate aftermath of high-dose radiation therapy, we have demonstrated attenuated bone loss in mechanically loaded limbs. The significant benefit loading imparted on irradiated bone warrants consideration of loading and underlying signaling pathways for post-irradiation/transplantation bone health and reduction of fracture risk.

## Supporting Information

S1 Fig Raw trabecular microstructural parameters analyzed by microCT. Presented as scattered dot plots with bars representing mean ± 95% CI, n = 6 for ‘Irradiated Mice’ and n = 10 for ‘Irradiated+Loaded Mice’. Within each graph, different letters indicate a significant difference and same letters indicate no difference by repeated measures one-way ANOVA with Tukey’s multiple comparisons test (p<0.05).
(TIF)

S2 Fig Raw cortical microstructural parameters analyzed by microCT. Presented as scattered dot plots with bars representing mean ± 95% CI, n = 6 for ‘Irradiated Mice’ and n = 10 for ‘Irradiated+Loaded Mice’. Within each graph, different letters indicate a significant difference and same letters indicate no difference by repeated measures one-way ANOVA with Tukey’s multiple comparisons test (p<0.05).
(TIF)
